# Operative Treatment of Facial Palsy

**Published:** 1903-05-16

**Authors:** 


### OPERATIVE TREATMENT OF FACIAL PALSY.

The deformity brought about by complete facial paralysis which has not proved amenable to time and medical treatment is a misery sufficient in itself to warrant surgical interference provided this can be carried out with a reasonable prospect of relief. In an interesting paper on the subject Messrs. C. A. Ballance, H. A. Ballance and Dr. Stewart [1] make some observations based on seven such cases which were submitted to operation. In six of these a junction was effected between the distal end of the facial nerve and the spinal accessory nerve, in the seventh a facio-hypoglossal anastomosis was performed. In this case and one of the others operation had been performed too recently to allow of any obvious improvement, in all the remaining five the results were satisfactory. In four of these patients the paralysis was due to chronic otitis media, while in the remaining one it followed fracture of the base of the skull. The duration of the lesion previous to operation varied from four and a half months to nearly three years. The principle of the treatment is founded on the fact that regeneration occurs to a certain extent in the distal segment of a divided nerve, and that such regeneration may become complete if the distal segment is joined to the proximal so as to permit of the passage of impulses. The authors consider that no interval of time is too long for attempted cure so long as any muscle fibres survive which may be innervated by the regenerated nerve, as shown by response to galvanic stimulation. The technique employed in their cases was as follows:—The facial nerve was exposed at its point of exit from the stylomastoid foramen and divided as high up as possible. The spinal accessory nerve was then exposed and its sheath incised, and the distal segment of the facial nerve inserted into it by means of fine silk sutures. After the wound was healed galvanism was applied daily to the paralysed muscles until faradic excitability had returned. The results were fairly uniform. The facial muscles recovered their contractility, but they could only be brought into action in conjunction with the muscles supplied by the spinal accessory nerve, and conversely whenever the muscles supplied by the latter nerve were brought into action the facial muscles also contracted. In one case it appeared as if power of dissociation might be eventually acquired.

[1] British Medical Journal, May 2nd.

